# A Suggestion or Aid to the Eradication of Tuberculosis by a Practical and Profitable Method

**Published:** 1895-10

**Authors:** A. S. Heath


### A SUGGESTION OR AID TO THE ERADICATION OF TUBERCULOSIS BY A PRACTICAL AND PROFITABLE METHOD.

BY A. S. HEATH, M.D., V S.

THERE are 16,500,000 of cows in the United States, of this vast number 8,000,000 are unprofitable. There are 2,000,000 susceptible to, or tuberculous. This is a bad showing for the dairy interest. The average cow yields 130 pounds of butter. It costs $35 to feed her, $12.50 for labor and care, and $2.50 interest on her investment. This makes $50. And 130 pounds of butter at 25 cents per pound, gives $32, from $50 her annual cost, shows $18 loss; and this multiplied by 8,000,000 cows, makes $144,000,000 loss. Add to this 2,000,000 of tuberculous at $30 per head, footing up with the $144,000,000 the total loss of $204,000,000, a bonded debt that never brings out a smile from the sad face of any farmer. The protection is of the crushing kind.

Unless we can get up to 300-pound butter cows, or its equivalent product, our farmers cannot make a profit on their capital and labor; 200-pound cows do not pay.

In the Normandy and spotted Swiss or Simmenthal blood we have three hopes: more and better butter, more and better beef, and less tuberculosis and less loss.

The 300-pound butter cow gives us $600,000,000 profit; the 400-pound, yields $800,000,000 profit.

Mrs. E. M. Jones, of Canada, made $49.70 from a good grade cow in a year. If the product had been sold as cream at 20 cents per quart, or two and a half times more, it would have brought her $124.25. And if the same result could have been realized throughout the United States with all our paying cows, it would have amounted to the encouraging sum of $994,000,000.

The annual average milk-yield from the cows of the United States, according to the late census, was 2883 pounds. The Norman and spotted Swiss or Simmenthal cross should yield three or four times as much. Should our cows be turned into beef at $50 per head, $400,000,000 could be realized; while the same number of the grade Normans should bring $600,000,000, for very much better beef at the same price.

The time is fast approaching when the best beef must be produced near the home of consumption. For ranch beef is too small, too poor, too difficult to get to market in the best possible condition to supply the constantly increasing demand for the best quality. And when the political millenium of "roast beef and two dollars a day" shall come, the demand for beef and dollars will be enormous by workingmen.

Everyone hopes for the coming of better times.

He who advocates the dissemination of the blood of a race or breed of animals, because of one or more superior excellences, should not forget that he also combines the excellences of all the breeds whose blood he purposely commingles. The Jersey possesses excellences that, united with and modifying those of the Norman and spotted Swiss or Simmenthal, must produce a progeny of surpassing value to either the Jersey or the Norman and spotted Swiss. So also the union of the choice strains of the Holstein, we know by experience, has produced a dairy-strain-combination of characteristic dairy value. I instance Mr. H. B. Gurler's herd. He says, in his excellent work on *Amer-*

*ican Dairying*, "I now have on my farm sixty-five heifers that are from grade Holstein-Friesian cows and registered Jersey bulls. They are a very promising lot of heifers, and I feel confident they will do me good work. They have the Jersey markings mostly, and are open and roomily built, with good size and large digestive organs."

I can assure Mr. Gurler, from personal experience, that good results will follow this blood combination of these noble breeds, if careful selection for the dairy was made from both. Some years since I wrote to the *Jersey Bulletin* on the utility of this combination from the value of the products of the cows thus bred.

I am advising the crossing of the Normandy and Jersey and Swiss Simmenthal for the valuable mutual modification of qualities and the accumulation of the excellences of all breeds. The anticipated result will doubtless be greater size, more stamina, larger freedom from tuberculosis, larger veal-calves, more and better veal and beef on the Jersey side; and the *fining* of the bone and skin, the beautifying of the head, and the enriching of the milk of the Norman and Swiss on the other. The possibilities of crosses and grades in largely augmenting the value of our herds and their products will, in a few years, far exceed the most ardent anticipations, I doubt not, that I or other shope to see realized.

My advice is cherish and improve the registered animals, and use them freely in crossing whenever accumulated excellences can be thus secured. For no one breed possesses all the excellences in a superlative degree. All excellence is relative. What are termed common breeds are not devoid of qualities of real value. These valuable qualities should be collected and concentrated in the cross-bred animals of the several breeds. The "common stock" is the cheapest foundation for collecting the superior excellences of all the breeds for special products. By this method the surplus males of the pure breeds can be profitably utilized. Above all, in breeding there is a stern necessity to exclude the worthless—*the animal weeds*—the delicate, the unhealthy. A long and choice pedigree should be accompanied invariably by an animal of surpassing excellence, sound health, and stamina. Relationship—family—is well enough provided the relation—the individual—possess in himself those sterling qualities that command recognition by fortified possession. The amount of pure gold in the coin fixes its value. The Normandy and spotted Swiss blood has so much of the pure gold-value in it that it will pass current in coining its excellences on the several breeds and common herds of our vast pasture-lands.

"There are millions in it!"

> "But words are things, and a small drop of ink,
> Falling, like dew, upon a thought, produces
> That which makes thousands, perhaps millions, think."

Look at the marvelous wealth diffused throughout the civilized world by means of the short-horn breed of cattle. Their blood has improved all the beef-producing herds whenever it has been diffused in any degree. It required more than a hundred years to bring it up to its present degree of great beef excellence. Individuals of this breed have been sold at public auction for more thousands of dollars than any other breed of cattle. Beef is their superlative excellence; and while to the English belongs the honor of originators, American breeders have stamped the genius of improvement alike on this and every other breed upon which they have laid their hands. The tests of the Jerseys, Guernseys, Holsteins, Ayrshires, all foreign breeds, have here been made to reach higher figures than were ever attained in their native homes. It is in view of these accomplishments that I confidently look to grander results than have yet been realized in our flocks and herds, both in their sum total of health and value of products.

> "Catch, then, O catch the transient hour;
> Improve each moment as it flies;
> Life's a short summer, a man a flower;
> He dies—alas! how soon he dies!"

